# Interactive and open educational resources for advanced EEG analysis

**DOI:** 10.1002/epd2.70209

**Published:** 2026-02-20

**Authors:** S. Beumer, I. Rigoni, S. Lagarde, S. Vulliemoz, N. Roehri

**Affiliations:** ^1^ EEG and Epilepsy Unit, University Hospitals and Faculty of Medicine University of Geneva Geneva Switzerland; ^2^ Assistance Publique‐Hôpitaux de Marseille Epileptology and Cerebral Rhythmology Department, Timone Hospital Marseille France; ^3^ Aix Marseille Univ INSERM, INS, Inst Neurosci Syst Marseille France; ^4^ Center for Biomedical Imaging Lausanne Switzerland

Advanced EEG analysis techniques are expected to play an increasingly prominent role in both clinical practice and neuroscientific research, given their potential for clinical diagnosis and monitoring,^1^ as well as for the discovery and validation of EEG‐based biomarkers in research settings.^2^, ^3^

Examples of such methods include electrical source imaging for the non‐invasive localization of the epileptogenic zone and functional connectivity analyses to characterize network‐level dysfunction. Although these techniques are increasingly reported in the clinical and cognitive neuroscience literature and implemented in research‐grade software, their underlying assumptions and limitations are often not immediately apparent to end users. In contrast to conventional visual inspection of EEG traces and voltage topographies, these approaches rely heavily on mathematical modeling, signal processing, and statistical inference. As a result, their correct application and interpretation resquire a level of methodological understanding that is not typically addressed in standard clinical EEG training and neuroscience courses.

This creates a growing need for educational resources that bridge the gap between theoretical principles and their application to EEG, both in clinical and in research settings. Viewed from an educational perspective, this gap is not merely technical but pedagogical, as abstract concepts such as inverse modeling or network metrics are difficult to grasp without interactive and data‐driven illustrations.

These considerations lead to educational approaches that enable the interactive exploration of advanced EEG concepts instead of passive learning through slides and formulas. Such tools are already in use to help learn how to interpret traces^4^ and label independent components.^5^ However, to our knowledge, no platform integrates the theory behind advanced EEG analysis methods with clinical‐data‐driven demonstrations and generative quizzes within an open‐source framework. To address this, we introduce a collection of interactive, browser‐based teaching tools for advanced EEG analysis designed to support students, clinicians, and researchers by leveraging real‐world case studies in epilepsy. However, the underlying methods are not limited to epilepsy and can be readily applied to other scenarios, such as evoked potentials, pathological activity, and resting‐state recordings.

Our web‐based application, hosted on GitHub* and in a Zenodo repository,^6^ is fully open source, ensuring transparency, reproducibility, and extensibility. Visualization relies on the Niivue framework^7^ as well as plotly.^8^

The platform is organized into three complementary sections: Courses, Demos, and Quizzes. Courses establish the mathematical foundations through interactive, hands‐on examples. Demos enable in‐depth exploration of real‐world case studies to develop intuition, and Quizzes provide procedurally generated exercises to evaluate and consolidate learning. The real‐world cases are taken from the epilepsy field, namely, localization of interictal epileptiform discharges, ictal or interictal connectivity based on intracranial EEG or high‐density EEG. Currently, the site includes instructional modules on source localization and functional connectivity. Each demo has an accompanying documentation page, accessible via the “About” button, that explains to the user how the parameters influence the visualization. A set of quizzes is provided to evaluate understanding of vector addition, correspondence between graph and matrix representations, dipole localization, and the methods used to generate the displayed source activity.

In Figure 1, several components of the developed web environment are illustrated. Panel (A) demonstrates how the phase‐locking value evolves as frequency, phase, and noise parameters are varied (see Courses: Phase‐based connectivity). Panel (B) allows users to inspect the voltage topography and the corresponding inverse solution during an interictal epileptiform discharge, while interactively adjusting settings such as the regularization parameter and visualization threshold (see Demos: Illustration of the impact of the regularisation…). Panel (C) shows the connectivity matrix between regions of interest derived from high‐density EEG. Users can adjust the visualization, connectivity metric, and frequency band to explore how these choices affect the resulting networks (see Demos: Group averaged non‐invasive interictal connectivity…). Finally, panel (D) provides a quiz interface in which users can test their knowledge and intuition by identifying the dipole corresponding to a given voltage map (See Quizzes: Topography Quiz).

**FIGURE 1:**
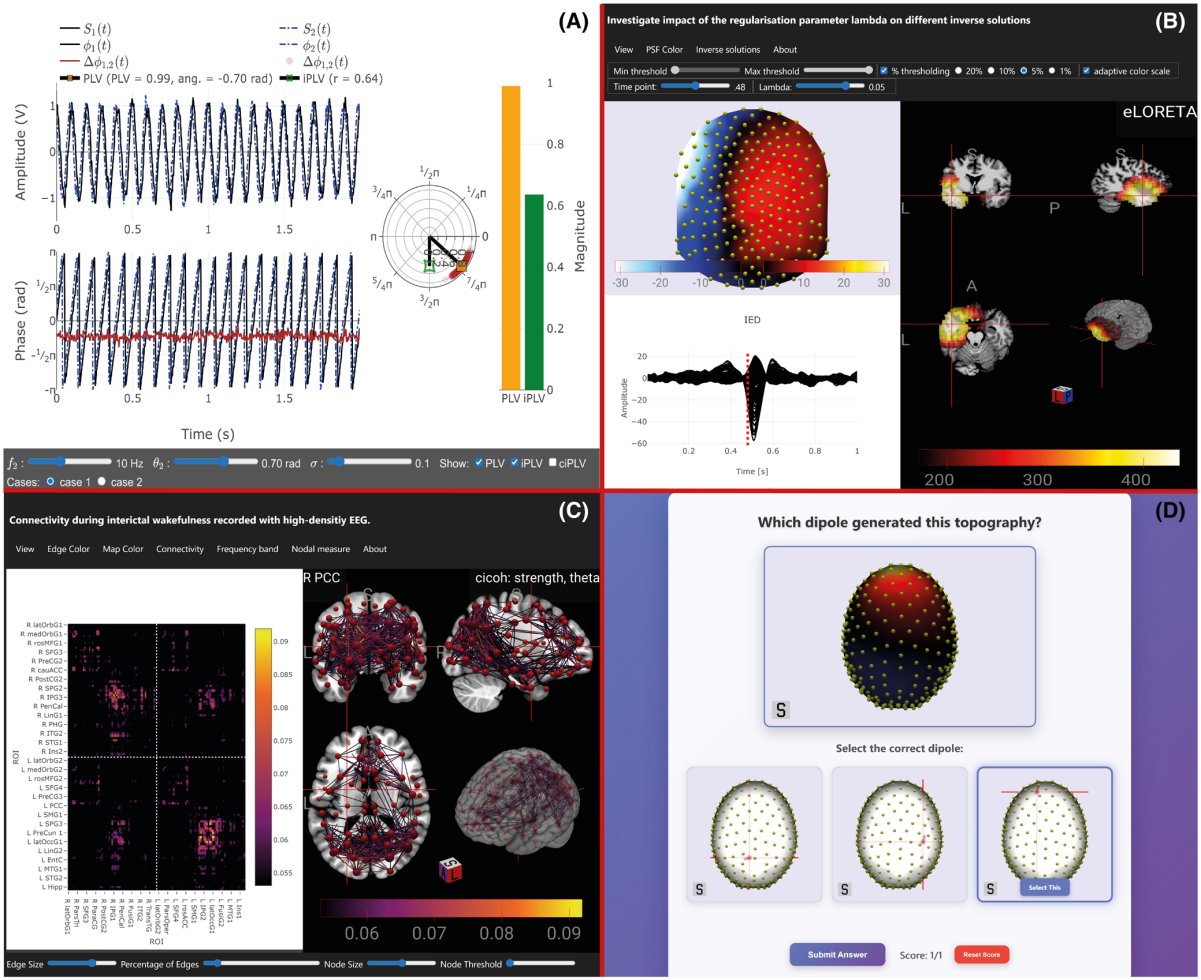
Overview of the educational materials and interactive demonstrations. (A) Example lecture page on phase‐based connectivity calculations, including the relevant mathematical formulations and illustrative examples using simple (amplitude‐modulated) sinusoids. (B, C) Interactive demonstrations based on clinical data: (B) allows the user to adjust parameters of the inverse solution for an interictal epileptiform discharge, while (C) displays a connectivity matrix alongside a three‐dimensional visualization (network resembling the default mode network shown). (D) Example quiz interface in which users can test their ability to identify the dipole corresponding to a given voltage topography.

We believe that this platform fills an important gap in the education of advanced EEG methods for neuroscientists and clinicians. Readers are encouraged to visit our GitHub repository to explore the full materials and contribute by suggesting improvements or adding new features, either through direct correspondence with the corresponding author or via the GitHub Issues system. Future extensions of the platform will include additional modalities and methods, such as MEG, power spectrum fitting, and time‐frequency analysis.

## CONFLICT OF INTEREST STATEMENT

Authors declare no conflicts of interest.

## Data Availability

The data that support the findings of this study are openly available in Interactive web‐based applications for teaching and learning at https://github.com/Roehrin/webapps_for_EEG_courses and https://zenodo.org/records/17966135, reference number https://doi.org/10.5281/zenodo.17966135.

## Footnotes

* See: https://roehrin.github.io/webapps_for_EEG_courses/.
